# Use of Radioactive Iodine in Type 2 Amiodarone-Induced Thyrotoxicosis

**DOI:** 10.1016/j.aed.2025.05.002

**Published:** 2025-05-08

**Authors:** Sonia Rodrigues, Run Yu

**Affiliations:** 1 Division of Endocrinology, UCLA David Geffen School of Medicine, Los Angeles, California; 2 Division of Endocrinology, Diabetes and Metabolism, Department of Medicine, Veterans Affairs Greater Los Angeles Healthcare System, Los Angeles, California

**Keywords:** amiodarone induced thyrotoxicosis, hyperthyroidism, radioactive iodine ablation, radioactive iodine uptake

## Abstract

**Background/Objective:**

Amiodarone-induced thyrotoxicosis (AIT) is a well-recognized etiology of hyperthyroidism. A case is reported here which demonstrates that radioactive iodine ablation therapy (RAI) can be used to treat type 2 AIT, even at lower radioactive iodine uptake (RAIU) levels than what conventional teaching would recommend for RAI.

**Case Presentation:**

An 80-year-old male with atrial fibrillation and cardiomyopathy was found to have type 2 AIT. He was refractory to other treatments for atrial fibrillation and therefore dependent on amiodarone. He was started on prednisone to treat AIT but developed fluid overload. As he was deemed a poor surgical candidate for thyroidectomy, recombinant human thyroid-stimulating hormone-stimulated RAI was administered with 29.5-mCi I-131; he subsequently developed subclinical hypothyroidism despite pre-RAI RAIU of 3%. He remained on amiodarone until he received a heart valve replacement, which temporarily relieved the atrial fibrillation and allowed for amiodarone discontinuation. After atrial fibrillation recurred and in anticipation of resuming amiodarone, he received a second dose of recombinant human thyroid-stimulating hormone-stimulated RAI at 26.5 mCi I-131, which rendered him clinically hypothyroid.

**Discussion:**

RAI therapy can be considered as a potential treatment strategy for type 2 AIT if the RAIU is at least 3%.

**Conclusion:**

This case demonstrates that RAI is an effective treatment strategy for type 2 AIT if the patient cannot tolerate steroids and is not a candidate for thyroidectomy. This case also illustrates how RAI can be used to prevent AIT.

**Highlights**

• Radioactive iodine ablation therapy (RAI) can be considered in patients with acute type 2 amiodarone-induced thyrotoxicosis (AIT)
• Thyrogen stimulation can be used to increase efficacy in RAI for type 2 AIT
• RAI can also be used to prevent recurrent type 2 AIT

**Clinical Relevance**

Radioactive iodine ablation therapy can be used as a treatment strategy for patients with acute type 2 amiodarone-induced thyrotoxicosis who are intolerant to corticosteroids and are poor candidates for thyroidectomy.

## Introduction

Amiodarone is a class III antiarrhythmic drug that is used to treat both atrial and ventricular arrhythmias. Thyroid toxicity is a well-known adverse effect of amiodarone due in large part to its high iodine load. For example, in a daily dose of 200-400 mg amiodarone, there are 6-12 mg of iodine, which far exceeds daily iodine requirements.^1^ For this reason, the American Thyroid Association recommends monitoring thyroid function tests at 3-to-6-month intervals for all patients started on amiodarone.^2^ The incidence of amiodarone-induced thyrotoxicosis (AIT) ranges from 5% to 10%.^3^ There are 2 mechanisms for AIT: the first is similar to the Jod-Basedow phenomenon that classically occurs in patients with a predisposition for hyperthyroidism; the second is destructive thyroiditis due to direct cytotoxicity of amiodarone on the thyroid follicular cells.^3^ AIT management usually involves switching to alternative anti-arrhythmic medications when feasible, as well as medical management with antithyroid drugs (for type 1) and corticosteroids (for type 2). If medical management is unsuccessful, thyroidectomy is considered. Radioactive iodine therapy (RAI) is not recommended for management of type 2 AIT in general but has been described in case reports and some series as a viable approach to managing type 2 AIT.^4^, ^5^, ^6^ A case is reported here describing a patient with type 2 AIT who was intolerant to corticosteroids and a poor candidate for thyroidectomy but was successfully managed by RAI.

## Case Report

An 80-year-old male was referred to endocrinology clinic for hyperthyroidism. He had a history of rheumatic heart disease requiring mitral valve replacement, hypertension, and atrial fibrillation for which he had been taking amiodarone for 3 years, with current dose of amiodarone 200 mg twice daily. Thyroid function was tested after he started to feel worsening dyspnea. Thyroid-stimulating hormone (TSH) level was <0.02 μIU/mL (normal range 0.3-4.7), free T4 (FT4) 3.3 ng/dL (0.8-1.7), and free T3 (FT3) 454 pg/dL (222-383); thyroid peroxidase antibody level was normal (Fig.). He had no family history of thyroid disease. He completed parathyroidectomy with partial left thyroidectomy 20 years prior for primary hyperparathyroidism and a thyroid nodule; surgical pathology revealed a parathyroid adenoma and small microfollicular thyroid adenoma. His TSH had been 1.6-2.4 in the previous 3 years. His weight was 82.2 kg. No thyroid abnormalities were found by physical examination, and he denied specific symptoms of hyperthyroidism. Ultrasound demonstrated mildly heterogenous echotexture of thyroid parenchyma with normal blood flow on Doppler. The right lobe measured 4.1 × 2.3 × 1.5 cm and the left lobe measured 4 × 2.6 × 1.8 cm; scattered subcentimeter cystic nodules were also noted.

**Fig:**
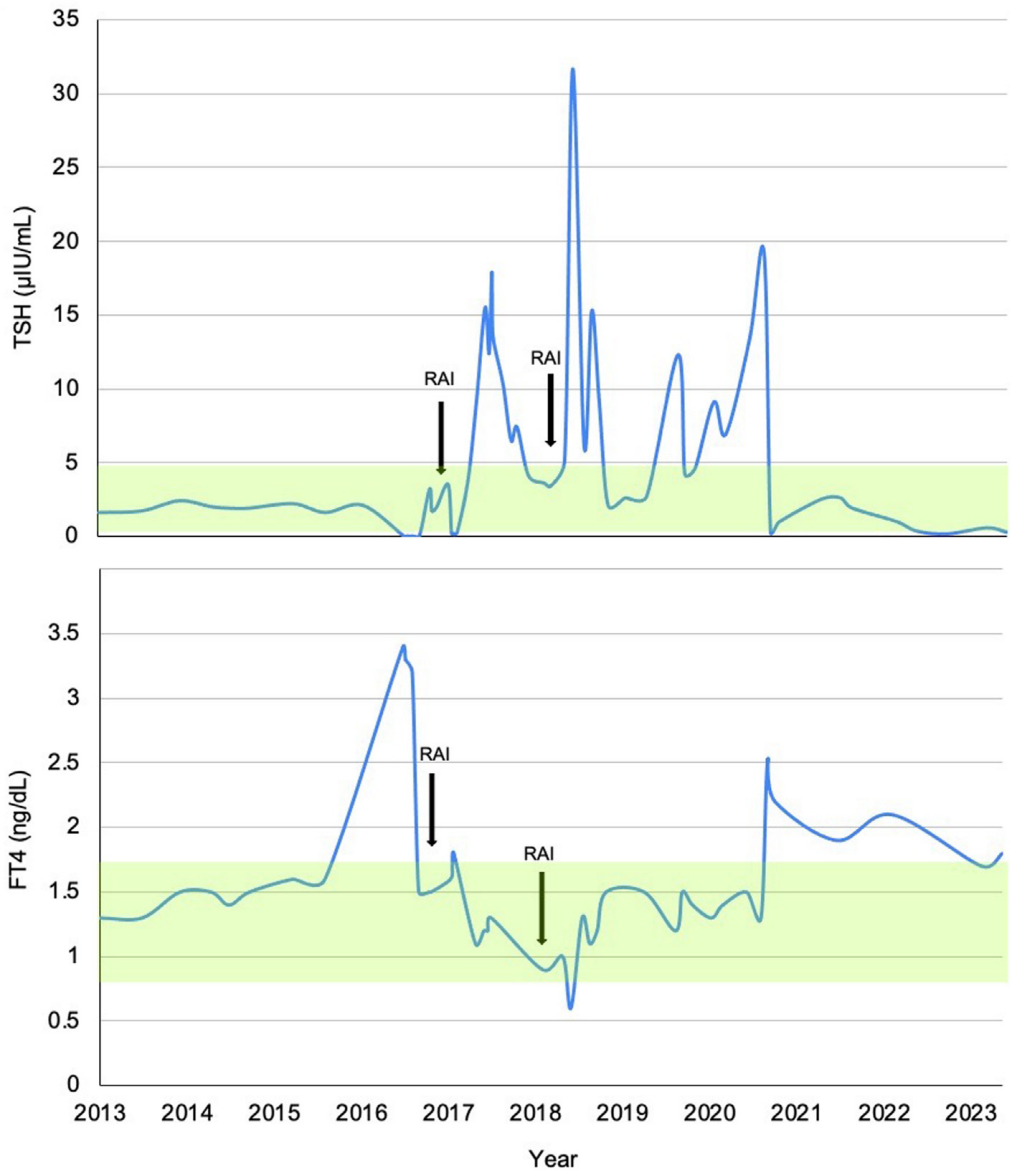
Serum thyroid-stimulating hormone (TSH) and free T4 (FT4) levels over time. The yellow highlighted region represents normal TSH and FT4 ranges. The arrows represent the 2 time points in which the patient received radioactive iodine ablation (RAI) therapy. The first arrow marks when the patient received RAI during an amiodarone-induced thyrotoxicosis (AIT) episode, and the second arrow marks when he received RAI to prevent recurrence of AIT.

The patient underwent a radioactive uptake (RAIU) and scan, which showed 2.0% uptake at 6 hours and 1.5% uptake at 24 hours with no significant tracer in either thyroid lobe. He was diagnosed as having type 2 AIT; he continued a beta blocker and started prednisone 40 mg daily with a plan to taper corticosteroids based on thyroid response. He responded well to prednisone with normalization of thyroid function tests but started to notice weight gain and dyspnea, which his cardiologist attributed to prednisone despite concurrent furosemide use. Of note, he continued using amiodarone for atrial fibrillation, which had proven refractory to other medication regimens, including dronedarone.

Thyroidectomy was deemed suboptimal given his multiple comorbidities and previous neck surgery. A decision was made to repeat RAIU to evaluate the potential for RAI. RAIU was performed 3 months after the initial diagnosis of hyperthyroidism while he was still taking prednisone 10 mg daily; uptake was 3.0% at 6 hours and 2.9% at 24 hours. He was then referred to an outside facility for RAI. Repeat RAIU at the outside facility showed 2.7% uptake at 4 hours and 3.4% uptake at 24 hours. He subsequently received recombinant human thyroid-stimulating hormone (rhTSH) 0.9 mg for 2 consecutive days before receiving RAI therapy with 29.5-mCi I-131 the following day; prednisone was stopped in the following week.

TSH initially decreased from 3.5 to 0.12 in the subsequent month with FT4 rising to 1.7. However, TSH started rising 9 weeks after RAI was administered. Three months after receiving RAI, TSH increased to 9.3 and FT4 decreased to 1.1 at which point the patient was instructed to start levothyroxine 25mcg daily as he was experiencing new-onset fatigue (Fig.). However, he reported palpitations with his first dose of levothyroxine and did not continue the medication. TSH rose as high as 17.9, but FT4 remained within normal limits. The patient underwent 3-valve replacement and shortly thereafter discontinued amiodarone; TSH normalized at that time without levothyroxine.

Unfortunately, the patient experienced recurrence of atrial fibrillation 6 months after his valve was repaired. At that point, he elected for prophylactic RAI in anticipation of needing amiodarone in the future. Repeat RAIU showed 7.3% uptake at 6 h and 11.7% uptake at 24 h. At that time, he had been off amiodarone for 9 months, and thyroid function testing was normal with TSH 3.6, FT4 0.9, and FT3 223. The following month he received rhTSH-stimulated RAI therapy with 26.5 mCi. Two months later, he developed hypothyroidism with TSH 31.7 and FT4 0.6 at which time he started levothyroxine 88mcg daily (Fig.). This dose was eventually titrated up to 125mcg daily, which was consistent with weight-based dosing; TSH normalized with that dose. Two years later, his cardiologist restarted him on low-dose amiodarone, after which he required higher doses of levothyroxine to maintain a euthyroid state.

## Discussion

The patient described here has clear type 2 AIT based on a suppressed TSH, elevated FT4 and FT3, and low RAIU after taking amiodarone for refractory atrial fibrillation. While the treatment for type 1 AIT hinges upon thionamide use, the hallmark for managing type 2 AIT is corticosteroids with a slow taper over 2 to 3 months. Additional medications to consider include lithium, iopanoic acid, and potassium perchlorate in type 1 AIT.^7^ On the other hand, mild cases of type 2 AIT may resolve spontaneously without medical therapies or stopping amiodarone.^2^ The rate of recurrence of type 2 AIT in patients who continue amiodarone treatment ranges from 6% to 75%.^3^ When medical treatment fails, thyroidectomy is the recommended intervention, particularly for patients with severe underlying cardiac disease. In the case of this patient with type 2 AIT, he was treated initially with a corticosteroid but could not continue it due to fluid retention; he was also a poor candidate for thyroidectomy given ongoing cardiomyopathy and arrhythmia history. Ultimately, he was successfully treated with RAI twice: first for type 2 AIT and later to prevent type 2 AIT recurrence.

Classically, RAIU is very low in cases of type 2 AIT, with uptake values < 3% due to the high iodine load and destructive processes of amiodarone; in contrast, RAIU uptake in AIT type 1 can be low, normal or even increased.^7^^,^^8^ In this patient, the initial RAIU was less than 3% at 6 and 24 hours, both because of type 2 AIT and concurrent amiodarone use. In order to be considered for RAI, RAIU typically must exceed 10%.^9^

Conventionally, RAI therapy is only considered for AIT once amiodarone is discontinued and urinary iodine levels normalize.^3^ RAI therapy, however, has been satisfactorily used for treatment of type 2 AIT in case reports and a few series. Notably, the overall efficacy varies depending on the RAI dose, whether rhTSH is given for the RAI, and duration of follow-up after RAI. One study looked at 79 patients who had developed AIT but remained on amiodarone. On average, RAIU in this group was 3.2% at 5 hours and 2.9% at 24 hours, but they still received 20 mCi of RAI. The study found that after 2 months, 2.5% of patients achieved a euthyroid state while 69.2% remained hyperthyroid.^1^ Another study showed that less than 15% of patients remain thyrotoxic 2 years after RAI therapy.^3^ These rates of persistent thyrotoxicosis after RAI therapy underscore why RAI should only be considered in difficult-to-treat cases. Fortunately, our patient developed subclinical hypothyroidism 3 months after the first RAI, allowing for continued amiodarone use and rendering corticosteroids unnecessary.

Based on case reports and usual treatment of type 2 AIT, RAI may be considered only if corticosteroid therapy fails and if the patient has a high surgical risk for total thyroidectomy, as in this patient's case. The reported RAI dose used in these cases ranges from 20-80 mCi, notably higher than those for Graves' disease or toxic thyroid nodules.^4^^,^^5^ This is due to the low RAIU in type 2 AIT. Although the use of rhTSH in RAI is controversial, its use may allow for lower RAI doses and help mitigate the risk of RAI-associated secondary malignancies. A case report looking at the management of a patient with Graves' disease who developed AIT during a cardiac episode demonstrated successful use of rhTSH and lithium to overcome low RAIU prior to treating with RAI. In that case, uptake increased from 6% to 32% at the 4-hour mark after receiving 2 doses of rhTSH.^10^ Patients receiving rhTSH for RAI should be monitored closely as they may develop severe thyrotoxicosis after RAI. A separate case report described a patient who received rhTSH with RAI for type 1 AIT and subsequently developed rising thyroid hormone levels and worsening atrial arrhythmia requiring ICU admission.^11^ As such, the European Thyroid Association recommends against rhTSH with RAI therapy for AIT.^10^ Although the risk of severe thyrotoxicosis after rhTSH-stimulated RAI for type 2 AIT should be lower, our patient also exhibited temporarily suppressed TSH without overt thyrotoxicosis following treatment. However, this more likely reflects the interval in which the patient was off prednisone and awaiting treatment effects of RAI therapy.

Per the 2016 American Thyroid Association guidelines, RAI should also be considered to prevent AIT. Specifically, it should be considered for patients with previous episodes of AIT and a history of life-threatening arrhythmias that only respond to amiodarone.^2^^,^^9^^,^^12^ While our patient initially received RAI therapy to treat type 2 AIT, he completed an additional round of RAI therapy in anticipation of resuming amiodarone in the future. Notably, the goal of RAI in these cases is to achieve a hypothyroid state, so as to prevent the recurrence of AIT for patients who must resume amiodarone.^9^

## Conclusion

This case demonstrates that RAI, though not typically used if RAIU is low, can be considered as a potential treatment for patients with type 2 AIT who cannot tolerate medical intervention with corticosteroids and would be poor surgical candidates for thyroidectomy.

## Disclosure

The authors have no conflicts of interest to disclose.
